# The Cumulative and Single Effect of 12 Aldehydes Concentrations on Cardiovascular Diseases: An Analysis Based on Bayesian Kernel Machine Regression and Weighted Logistic Regression

**DOI:** 10.31083/j.rcm2506206

**Published:** 2024-06-03

**Authors:** Yuemei Fang, Juan Zhang

**Affiliations:** ^1^Medical Imaging Department, Nanjing Brain Hospital, 210029 Nanjing, Jiangsu, China; ^2^Catheter Room of Cerebrovascular Disease Treatment Center, Nanjing Brain Hospital, 210029 Nanjing, Jiangsu, China

**Keywords:** Aldehyde, cardiovascular diseases, benzaldehyde, Bayesian kernel machine regression, weighted logistic regression

## Abstract

**Background:**

This study investigates the individual and cumulative effects of 12 aldehydes concentrations on cardiovascular disease (CVD).

**Methods:**

A total of 1529 individuals from the 2013–2014 National Health and Nutrition Examination Survey were enrolled. We assessed serum concentrations of 12 aldehydes, including benzaldehyde, butyraldehyde, crotonaldehyde, decanaldehyde, heptanaldehyde, hexanaldehyde, isopentanaldehyde, nonanaldehyde, octanaldehyde, o-tolualdehyde, pentanaldehyde, and propanaldehyde. CVD patients were identified based on self-reported disease history from questionnaires. The Bayesian kernel machine regression was used to evaluate the cumulative effect of 12 aldehyde concentrations on CVD. Both weighted and unweighted logistic regression were used to assess the association of serum aldehyde concentrations with CVD, presenting effect sizes as odds ratio (OR) with 95% confidence interval (CI). Additionally, a restricted cubic spline analysis was also conducted to explore the relationship between benzaldehyde and CVD.

**Results:**

Among the participants, 111 (7.3%) were identified as having CVD. Isopentanaldehyde concentrations were notably higher in CVD patients compared to those without CVD. Bayesian kernel machine regression indicated no cumulative effect of aldehydes on CVD. Unweighted logistic regression revealed a positive association between benzaldehyde and CVD when adjusting for age and sex (OR = 1.12, 95% CI = 1.03–1.21). This association persisted after adjusting for age, sex, race, education, hypertension, diabetes, alcohol consumption, and smoking, with an OR of 1.12 (95% CI = 1.02–1.22). The restricted cubic spline showed a linear association between benzaldehyde and CVD. In the weighted logistic model, the association between benzaldehyde and CVD remains significant (OR = 1.17, 95% CI = 1.06–1.29). However, no significant association was found between other aldehydes and CVD.

**Conclusions:**

Our study reveals the potential contributing role of benzaldehyde to CVD. Future studies should further validate these findings in diverse populations and elucidate the underlying biological mechanisms.

## 1. Introduction

Aldehydes, a class of organic compounds characterized by a carbonyl group bonded to at least one hydrogen molecule [1], are ubiquitous in the environment. Aldehydes originate from diverse sources such as tobacco smoke, environmental pollutants, food consumption, and endogenous biological pathways [2, 3, 4]. Recent research has increasingly highlighted the detrimental effect of aldehydes on human health, leading to various health complications. Elevated hexanaldehyde levels, for instance, were reported to be associated with nasal obstruction and mild irritation, causing symptoms like frequent eye blinking and headaches [5]. Furthermore, the carcinogenic and mutagenic characteristics of aldehydes have been extensively documented [6]. Studies have found increased serum levels of hexanaldehyde and heptanaldehyde in patients with lung cancer [7], and higher concentrations of pentanaldehyde, nonanaldehyde, hexanaldehyde, and octanaldehyde in exhaled breath of patients with lung cancer [8].

The heart and blood vessels demonstrate increased sensitivity following aldehyde exposure [9, 10]. Studies have shown that aldehydes present in the blood vessel wall could induce hypercontraction, elevate the risk of vasospasm, and potentially result in myocardial necrosis [10]. Additionally, aldehydes are known to cause inflammation in blood vessels, promote intravascular thrombosis, and disturb the production of nitric oxide (NO) in vascular endothelial cells. This impairment of NO-mediated endothelial function and the disruption of NO’s cardioprotective effects may increase the risk of coronary artery disease [11]. Although the precise mechanisms are not yet fully understood, growing evidence suggests a potential link between aldehydes and cardiovascular diseases (CVD) [12, 13].

Given the widespread environmental exposure to aldehydes, there is growing concern about their adverse effects on the cardiovascular system. This study, therefore, aims to investigate the relationship between the concentrations of 12 specific aldehydes and CVD based on the representative population data.

## 2. Methods

### 2.1 Participants Sources

Participants for this study were sourced from the National Health and Nutrition Examination Survey (NHANES), an initiative of the National Center for Health Statistics under the Centers for Disease Control and Prevention [14]. NHANES is a comprehensive, nationally representative survey that assesses the health and nutritional status of the U.S. population through interviews and physical examinations. The survey applied a complex stratified multistage-clustered sampling design to ensure national representation. The National Center for Health Statistics Ethics Review Board approved the NHANES survey (#2011-17), and written consent was obtained from all participants. For this study, we focused on the 2013–2014 NHANES data, as it was the only period during which aldehyde testing was conducted.

**Fig. 1.**
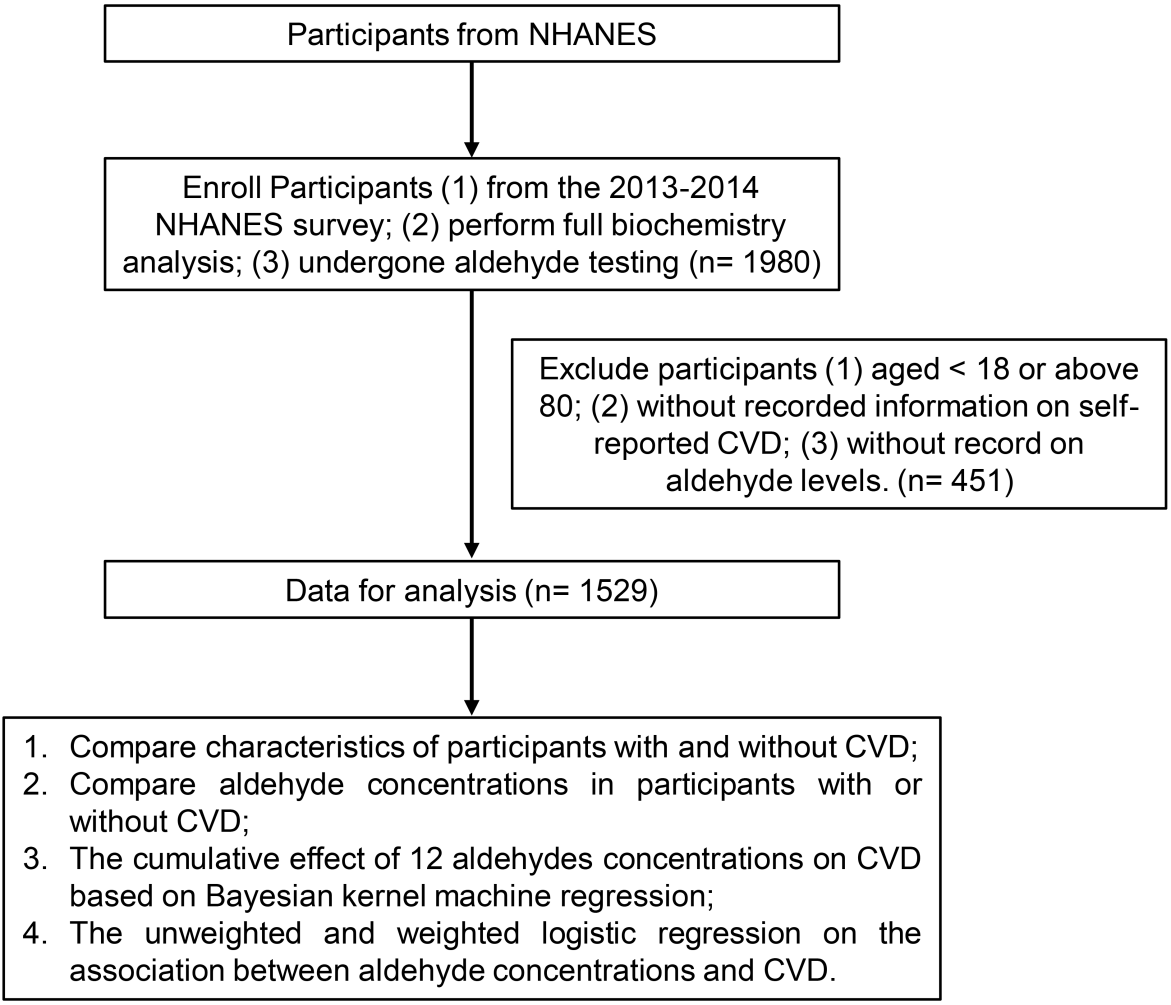
**The flow chart of the study.** CVD, cardiovascular disease; NHANES, National Health and Nutrition Examination Survey.

In this study, our inclusion criteria for participants from the NHANES survey were: (1) participants from the 2013–2014 NHANES survey; (2) completion of a complete biochemistry analysis; and (3) undergone aldehyde testing, which allowed us to analyze the concentrations of 12 specific aldehydes (benzaldehyde, butyraldehyde, crotonaldehyde, decanaldehyde, heptanaldehyde, hexanaldehyde, isopentanaldehyde, nonanaldehyde, octanaldehyde, o-tolualdehyde, pentanaldehyde, and propanaldehyde). The exclusion criteria included: (1) aged below 18 or above 80; (2) lack of self-reported CVD; (3) absence of aldehyde level records. Finally, 1529 individuals were enrolled in this study (Fig. 1).

### 2.2 Measurement of Serum Aldehyde Concentrations

The 2013–2014 NHANES survey employed a sophisticated automated analysis technique to measure the concentrations of 12 specific aldehydes: benzaldehyde, butyraldehyde, crotonaldehyde, decanaldehyde, heptanaldehyde, hexanaldehyde, isopentanaldehyde, nonanaldehyde, octanaldehyde, o-tolualdehyde, pentanaldehyde, and propanaldehyde. This technique involved solid-phase microextraction, gas chromatography, and high-resolution mass spectrometry, combined with isotope-dilution and selective ion mass detection methods. This approach can detect trace quantities of various aldehydes derived from protein adducts in human serum. Given that aldehydes commonly react with biological molecules to form products like Schiff base protein adducts, the NHANES survey specifically examines free aldehydes released from these adducts under acidic conditions (approximately pH 3). The automated process facilitated the breakdown of chemically bonded aldehyde adducts to proteins, allowing samples to be incubated with hydrochloric acid before analysis. This method utilizes isotope dilution to accurately measure minute quantities of aldehydes, boasting detection limits in the low parts per trillion. A comprehensive description of these serum aldehyde evaluation techniques is available in the Serum Aldehydes Laboratory Procedure Manual (https://wwwn.cdc.gov/nchs/data/nhanes/2013-2014/labmethods/ALD_ALDS_H_MET.pdf).

### 2.3 Identification of Cardiovascular Diseases

In the NHANES survey, trained interviewers conducted a series of questionnaires using a computer-assisted personal interviewing system. Participants were identified as having CVD based on self-reports of any of five cardiovascular outcomes: coronary heart disease, angina pectoris, heart attack, congestive heart failure, and stroke. In the questionnaires assessing medical conditions, the interviewers asked the following five questions: “Ever told you had coronary heart disease/angina pectoris/heart attack/congestive heart failure/stroke?”. Besides, the interviewers also provide explanations for these cardiovascular outcomes (shown in Table 1). Participants who responded ‘Yes’ to any of these questions were classified as having CVD [12].

**Table 1.** **The description for each cardiovascular disease**.

Disease	Description
Coronary heart disease	Is when the blood vessels that bring blood to the heart muscle become narrow and hardened due to plaque. Plaque buildup is called atherosclerosis. Blocked blood vessels to the heart can cause chest pain or a heart attack.
Angina pectoris	Angina is chest pain or discomfort that occurs when the heart does not get enough blood.
Heart attack	A heart attack happens when there is narrowing of a blood vessel that supplies the heart. A blood clot can form and suddenly cut off the blood supply to the heart muscle. This damage causes crushing chest pain that may also be felt in the arms or neck. There can also be nausea, sweating, or shortness of breath.
Congestive heart failure	Is when the heart can’t pump enough blood to the body. Blood and fluid “back up” into the lungs, which makes you short of breath. Heart failure causes fluid buildup in and swelling of the feet, legs and ankles.
Stroke	Is when the blood supply to a part of the brain is suddenly cut off by a blood clot or a burst blood vessel in the brain. The part of the brain affected can no longer do its job. There can be numbness or weakness on one side of the body; trouble speaking or understanding speech; loss of eyesight; trouble with walking, dizziness, loss of balance or coordination; or severe headache.

The descriptions for each cardiovascular disease were acquired from https://wwwn.cdc.gov/nchs/data/nhanes/2013-2014/questionnaires/MCQ_H.pdf.

### 2.4 Covariates

The NHANES survey collected demographic and health-related information through questionnaires, including age, sex, race (non-Hispanic White, non-Hispanic Black, Mexican American, other Hispanic, and other races), and education levels (below high school, high school, and above high school) were collected by questionnaires. Body mass index was measured by weight/(${\mathrm{height}}^{2}$). Cholesterol was collected from the standard biochemistry profiles.

Additionally, participants’ health conditions and habits were assessed. Those who self-reported having diabetes or hypertension were classified as having these conditions. Smoking status was determined by a history of smoking at least 100 cigarettes in their lifetime. Similarly, participants who had consumed at least 12 alcoholic drinks in the past year were categorized as drinkers.

### 2.5 Bayesian Kernel Machine Regression

The evaluation of the combined impact of multiple contaminants is crucial when analyzing complex environmental exposures. Bayesian Kernel Machine Regression (BKMR) stands out as an innovative and effective method for addressing this issue [15, 16]. In contrast to traditional regression techniques, which typically assume a linear and independent association between each contaminant and the health outcome, BKMR accommodates flexible, non-linear relationships and can detect interactive effects among the contaminants. It operates on the principle of using a kernel function to capture similarities between exposure profiles, integrating this within a Bayesian hierarchical framework. This approach not only quantifies uncertainty in the exposure-response relationship but also enables the identification of potentially harmful combinations or levels. For this study, the BKMR method was employed to analyze the cumulative effects of 12 aldehydes on CVD. This method is advantageous for modeling the complex interactions of these aldehydes, providing insights that simpler models might overlook. In this model, age and sex were considered as adjustment factors.

### 2.6 Statistical Analysis

In this study, continuous variables were presented as mean ± standard deviation (normal distribution) or median with Q1–Q3 (skewed distribution), and categorical variables were presented as frequencies. For comparing continuous variables, we used the one-way analysis of variance (ANOVA) test or the Kruskal-Wallis test, depending on the data distribution. Categorical variables were compared using the chi-square test. Additionally, we applied the weighted Kruskal-Wallis test to compare the aldehyde concentrations in participants with or without CVD. The sample weight of WTALD2YR was applied in the weighted statistics.

We used logistic regression to evaluate the association between the concentrations of 12 aldehydes and CVD. Two models were constructed: Model 1 adjusted for age and sex; and Model 2 adjusted for age, sex, race, education, hypertension, diabetes, drinking and smoking. A restricted cubic spline was also created to explore the association between benzaldehyde and CVD. Moreover, considering the sample design, weighted logistic regression was also applied. All statistical analyses were performed using R software (Version 4.1.1. R Foundation for Statistical Computing, Vienna, Austria).

## 3. Results

### 3.1 Participants Characteristic

Among the 1529 individuals, 111 (7.3%) were identified as patients with CVD. The participants’ characteristics are displayed in Table 2. There was a marked age difference between groups, with those in the CVD group having a median age of 64 years (Q1–Q3, 54.0–71.5 years) compared to a median age of 43 years (Q1–Q3, 30.0–59.0 years) in the non-CVD group. We observed a significant sex disparity in the CVD group, with a higher prevalence in males, accounting for 65.8% of CVD cases compared to 48.5% in the overall participants. Body mass index was significantly higher in the CVD group than in the control group. The prevalence of diabetes and hypertension was significantly higher in the CVD group (35.1% and 66.7%, respectively). The prevalence of smoking was significantly higher in CVD cases (64.0%), while there was no notable difference in drinking habits between the groups.

**Table 2.** **Participants characteristics**.

		All participants	Participants with CVD	Participants without CVD	*p*
		N = 1529	N = 111	N = 1418	
Age		45.0 (31.0, 60.0)	64.0 (54.0, 71.5)	43.0 (30.0, 59.0)	<0.001
Sex (male, n, %)		742 (48.5%)	73 (65.8%)	669 (47.2%)	<0.001
Race (n, %)					0.110
	Non-Hispanic White	680 (44.5%)	58 (52.3%)	622 (43.9%)	
	Non-Hispanic Black	256 (16.7%)	23 (20.7%)	233 (16.4%)	
	Mexican American	245 (16.0%)	11 (9.9%)	234 (16.5%)	
	Other Hispanic	134 (8.8%)	6 (5.4%)	128 (9.0%)	
	Other races	214 (14.0%)	13 (11.7%)	201 (14.2%)	
Education (n, %)					0.003
	Below high school	301 (20.9%)	34 (30.6%)	267 (20.1%)	
	High school	312 (21.7%)	30 (27.0%)	282 (21.2%)	
	Above high school	828 (57.5%)	47 (42.3%)	781 (58.7%)	
Cholesterol		4.8 (4.2, 5.5)	4.5 (3.8, 5.5)	4.8 (4.2, 5.5)	0.008
BMI (Kg/$m^{2}$)		27.7 (24.0, 32.5)	28.4 (25.9, 33.2)	27.6 (23.9, 32.4)	0.031
Diabetes (Yes, n, %)		160 (10.5%)	39 (35.1%)	121 (8.5%)	<0.001
Hypertension (Yes, n, %)		507 (33.2%)	74 (66.7%)	433 (30.5%)	<0.001
Smoking (Yes, n, %)		653 (42.7%)	71 (64.0%)	582 (41.0%)	<0.001
Drinking (Yes, n, %)		159 (10.4%)	10 (9.0%)	149 (10.5%)	0.736

CVD, cardiovascular disease; BMI, body mass index.

### 3.2 Aldehyde Concentrations in Participants with or without CVD

Table 3 shows the aldehyde concentrations in participants with or without CVD. Isopentanaldehyde showed a significantly higher concentration in participants with CVD (median value = 0.5 ng/mL; Q1–Q3, 0.4–0.7 ng/mL) than those without CVD (median value = 0.4 ng/mL; Q1–Q3, 0.3–0.7; *p* = 0.004). However, we observed on significant differences between the two groups in the other aldehyde concentrations, including benzaldehyde, butyraldehyde, crotonaldehyde, decanaldehyde, heptanaldehyde, hexanaldehyde, nonanaldehyde, octanaldehyde, o-Tolualdehyde, pentanaldehyde, and propanaldehyde. Consistently, the weighted Kruskal-Wallis test showed a similar trend, which indicated that the concentration of isopentanaldehyde was significantly higher in the CVD group (weighted *p* = 0.008).

**Table 3.** **Aldehyde concentrations in participants with or without CVD**.

	Participants with CVD	Participants without CVD	Unweighted *p*	Weighted *p*
	N = 111	N = 1418		
Benzaldehyde (ng/mL)	1.6 (0.9, 2.2)	1.4 (0.9, 2.0)	0.306	0.212
Butyraldehyde (ng/mL)	0.5 (0.2, 0.8)	0.5 (0.4, 0.7)	0.984	0.593
Crotonaldehyde (ng/mL)	0.1 (0.1, 0.2)	0.1 (0.1, 0.2)	0.875	0.322
Decanaldehyde (ng/mL)	2.8 (2.8, 2.8)	2.8 (2.8, 2.8)	0.147	0.333
Heptanaldehyde (ng/mL)	0.5 (0.4, 0.6)	0.5 (0.4, 0.6)	0.083	0.060
Hexanaldehyde (ng/mL)	2.0 (1.7, 2.6)	2.1 (1.8, 2.6)	0.391	0.432
Isopentanaldehyde (ng/mL)	0.5 (0.4, 0.7)	0.4 (0.3, 0.7)	0.004	0.008
Nonanaldehyde (ng/mL)	1.9 (1.9, 2.8)	1.9 (1.9, 3.1)	0.283	0.155
Octanaldehyde (ng/mL)	0.5 (0.5, 0.5)	0.5 (0.5, 0.5)	0.983	0.372
o-Tolualdehyde (ng/mL)	0.1 (0.1, 0.1)	0.1 (0.1, 0.1)	0.967	0.660
Pentanaldehyde (ng/mL)	0.2 (0.2, 0.4)	0.2 (0.2, 0.4)	0.212	0.688
Propanaldehyde (ng/mL)	2.0 (1.5, 2.6)	2.0 (1.5, 2.5)	0.634	0.072

CVD, cardiovascular disease.

### 3.3 The Cumulative Effect of 12 Aldehydes Concentrations on CVD Based on Bayesian Kernel Machine Regression

**Fig. 2.**
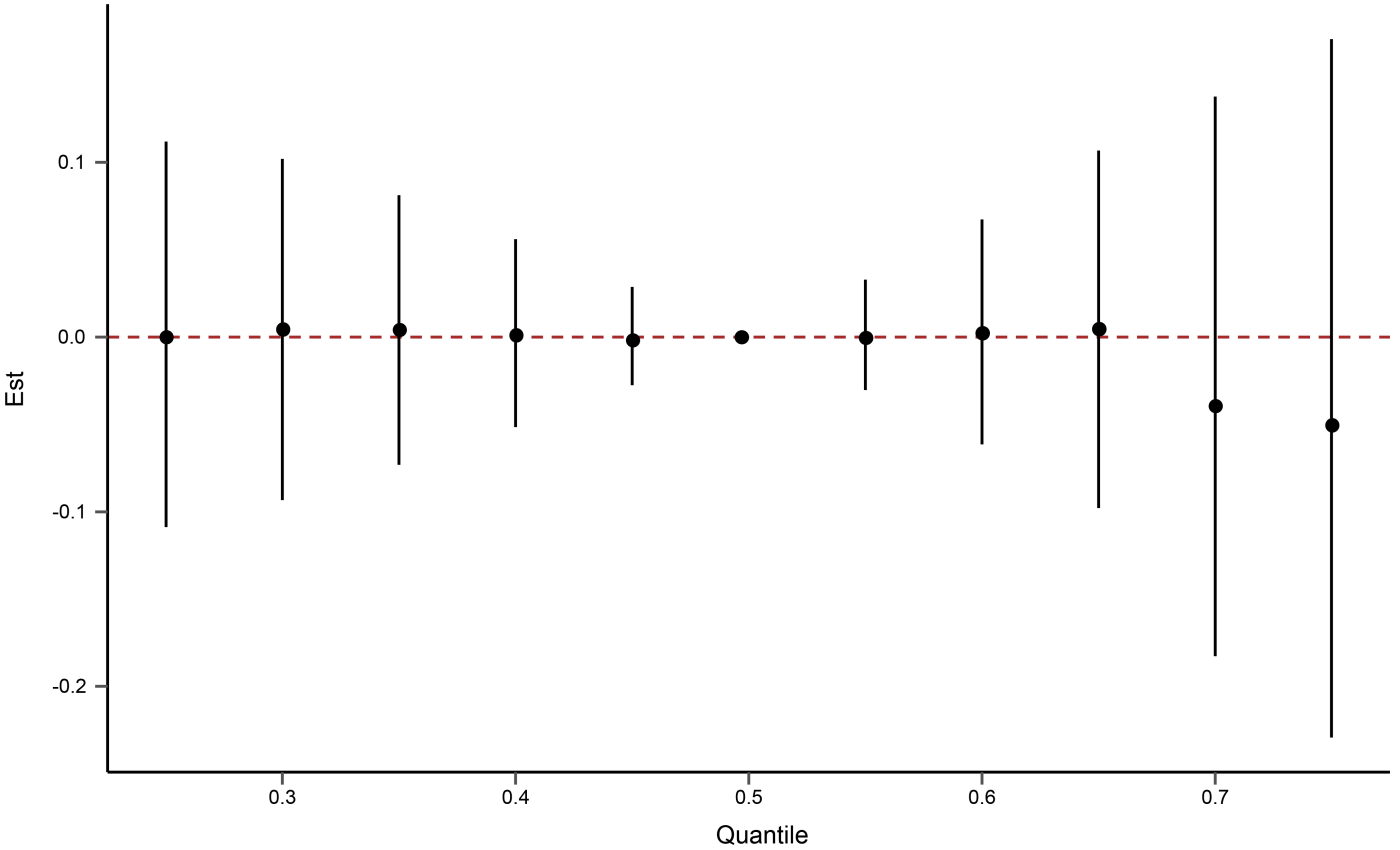
**The cumulative effect of the 12 aldehydes on cardiovascular diseases using Bayesian kernel machine regression.** Aldehydes exposures are at a particular percentile (X-axis) compared to the 50th percentile concentration.

The Bayesian kernel machine regression was used to evaluate the cumulative effect of 12 aldehydes on CVD. As shown in Fig. 2, no cumulative effect of aldehydes on CVD was observed.

### 3.4 The Unweighted Logistic Regression on the Association between Aldehyde Concentrations and CVD

**Fig. 3.**
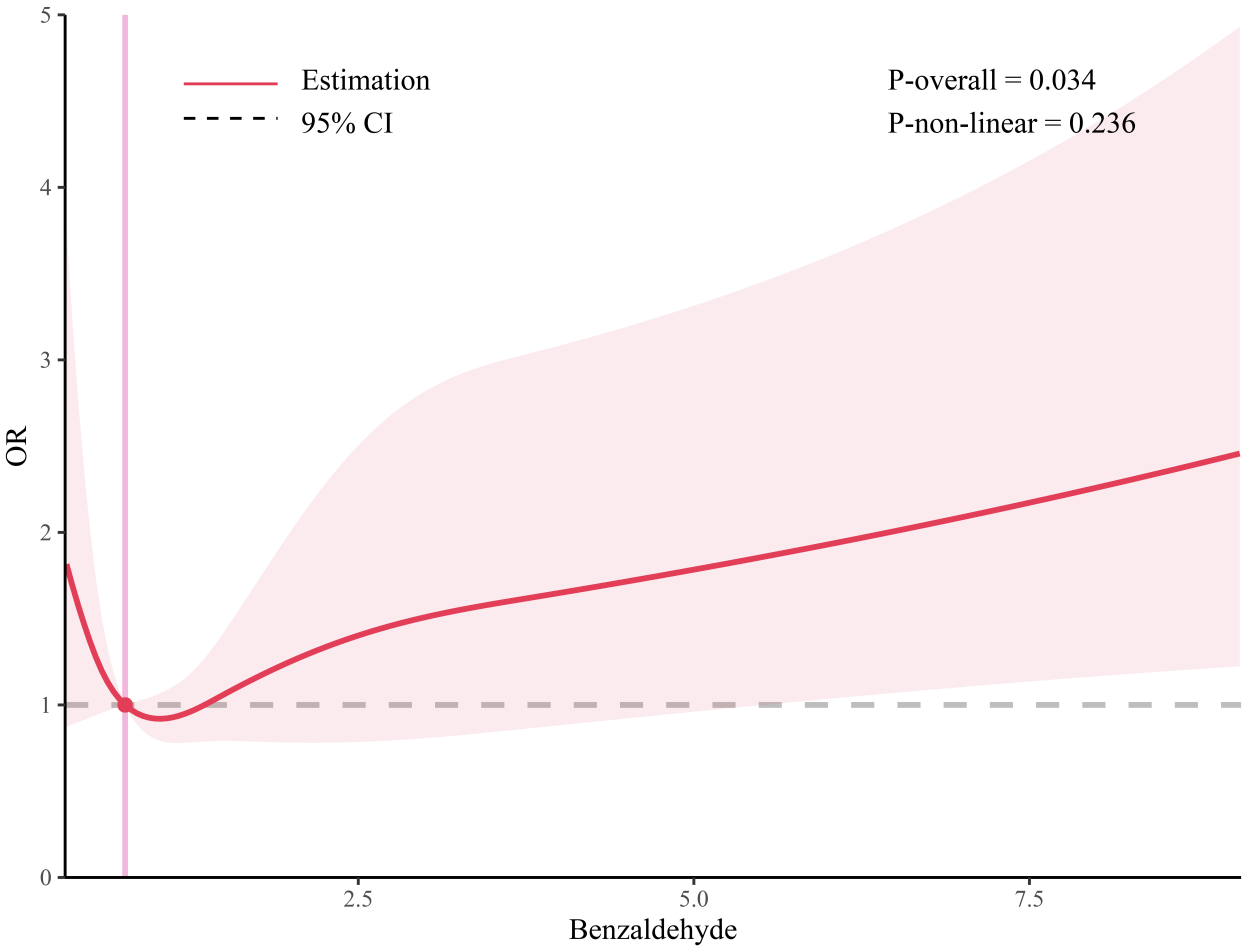
**The restricted cubic spline for the association between benzaldehyde and CVD. **CVD, cardiovascular disease; OR, odds ratio.

The results of logistic regression models investigating the potential relationship between the concentrations of each of the 12 aldehydes and CVD are displayed in Table 4. In the unweighted logistic regression, benzaldehyde showed a positive association with CVD when adjusted for age and sex (odds ratio (OR) = 1.12, 95% CI = 1.03–1.21). When the age, sex, race, education, hypertension, diabetes, drinking and smoking were adjusted for, the OR (95% CI) of benzaldehyde was 1.12 (1.02–1.22). Although isopentanaldehyde showed a significant association with CVD in model 1 (OR = 1.48, 95% CI = 1.01–2.12), no significant association was observed when adjusted for age, sex, race, education, hypertension, diabetes, drinking and smoking (OR = 1.49, 95% CI = 1.01–2.16). However, there were no significant associations for butyraldehyde, crotonaldehyde, decanaldehyde, heptanaldehyde, hexanaldehyde, nonanaldehyde, octanaldehyde, o-tolualdehyde, pentanaldehyde, and propanaldehyde. Additionally, the linear association between benzaldehyde and CVD is shown in Fig. 3.

**Table 4.** **The adjusted logistic regression between aldehyde concentrations and CVD**.

	Model 1			Model 2		
	OR	95% CI	*p*	OR	95% CI	*p*
Benzaldehyde	1.12	(1.03, 1.21)	0.003	1.12	(1.02, 1.22)	0.011
Butyraldehyde	1.37	(0.75, 2.37)	0.278	1.38	(0.74, 2.49)	0.295
Crotonaldehyde	0.52	(0.05, 2.94)	0.525	0.53	(0.05, 3.19)	0.556
Decanaldehyde	1.31	(0.58, 1.94)	0.295	1.46	(0.64, 2.16)	0.140
Heptanaldehyde	0.44	(0.09, 1.88)	0.299	0.56	(0.11, 2.34)	0.467
Hexanaldehyde	1.01	(0.87, 1.09)	0.785	1.02	(0.87, 1.09)	0.772
Isopentanaldehyde	1.48	(1.01, 2.12)	0.041	1.28	(0.82, 1.93)	0.252
Nonanaldehyde	0.91	(0.72, 1.10)	0.388	0.90	(0.70, 1.10)	0.352
Octanaldehyde	1.08	(0.29, 2.99)	0.890	1.07	(0.28, 3.12)	0.908
o-Tolualdehyde	0.23	(0.01, 8.81)	0.618	0.64	(0.01, 13.65)	0.889
Pentanaldehyde	1.47	(0.73, 2.45)	0.166	1.52	(0.71, 2.58)	0.146
Propanaldehyde	1.11	(0.90, 1.36)	0.316	1.1	(0.88, 1.36)	0.389

OR, odds ratio; CI, confidence interval; CVD, cardiovascular disease. Model 1 adjusted for age and sex. Model 2 adjusted for age, sex, race, education, hypertension, diabetes, smoking and drinking.

### 3.5 The Weighted Logistic Regression on the Association between Aldehyde Concentrations and CVD

We performed the weighted logistic regression to further explore the association between aldehyde concentrations and CVD. When adjusting for age, sex, race, education, hypertension, diabetes, smoking and drinking, we observed that the concentrates of benzaldehyde were significantly associated with CVD (OR = 1.17, 95% CI = 1.06–1.29). However, there was no significant association between other aldehydes and CVD in model 2. The results of weighted logistic regression are displayed in Table 5.

**Table 5.** **The weighted logistic regression between aldehyde concentrations and CVD**.

	Model 1			Model 2		
	OR	95% CI	*p*	OR	95% CI	*p*
Benzaldehyde	1.17	(1.07, 1.27)	0.002	1.17	(1.04, 1.32)	0.021
Butyraldehyde	1.16	(0.68, 1.98)	0.566	1.13	(0.56, 2.28)	0.650
Crotonaldehyde	1.01	(0.30, 3.40)	0.983	0.86	(0.15, 4.99)	0.826
Decanaldehyde	1.48	(0.92, 2.37)	0.098	1.68	(0.98, 2.87)	0.057
Heptanaldehyde	0.48	(0.10, 2.40)	0.339	0.48	(0.04, 5.57)	0.452
Hexanaldehyde	1.01	(0.94, 1.09)	0.662	1.02	(0.93, 1.11)	0.582
Isopentanaldehyde	1.60	(0.96, 2.67)	0.066	1.44	(0.68, 3.04)	0.249
Nonanaldehyde	0.82	(0.63, 1.07)	0.130	0.80	(0.53, 1.21)	0.209
Octanaldehyde	1.59	(0.41, 6.11)	0.470	1.50	(0.22, 10.36)	0.593
o-Tolualdehyde	1.45	(0.05, 42.07)	0.814	2.02	(0.08, 48.82)	0.572
Pentanaldehyde	1.55	(1.04, 2.32)	0.036	1.49	(0.90, 2.47)	0.095
Propanaldehyde	1.26	(1.03, 1.54)	0.029	1.24	(0.98, 1.57)	0.063

OR, odds ratio; CI, confidence interval; CVD, cardiovascular disease. Model 1 adjusted for age and sex. Model 2 adjusted for age, sex, hypertension, diabetes, smoking and drinking.

## 4. Discussion

This study investigated the relationship between aldehyde concentrations and CVD in 1529 participants from the 2013–2014 NHANES survey. To our knowledge, this is the first study to explore the association accounting for sample weights. Among the participants, those with CVD displayed a higher level of isopentanaldehyde than those without, though no significant differences were observed in the concentrations of the other 11 aldehyde types. In the unweighted logistic regression analysis, benzaldehyde concentration was significantly associated with CVD, showing an OR of 1.12 with 95% CI of 1.02–1.21, after adjusting for age, sex, race, education, hypertension, diabetes, smoking, and drinking. The significant association persisted in the weighted logistic regression analysis, with an OR (95% CI) of 1.17 (1.04–1.32). However, the levels of the other aldehydes did not show a significant association with CVD.

Aldehydes, a diverse class of organic compounds, are characterized by a carbonyl group where the carbon atom is bonded to a hydrogen atom and, typically, to another carbon or a hydrogen atom [17]. These compounds are omnipresent in nature and are commonly found in many foods, fragrances, and biological systems. Human exposure to aldehydes arises from multiple sources, such as air pollution, consumption of tobacco cigarettes and e-cigarettes, exposure to organic material, ingestion of food additives, alcohol intake, and endogenous metabolic activities [18, 19]. For example, crotonaldehyde and acrolein are significant aldehyde components of tobacco smoke. Primary exposure to these aldehydes in individuals occurs through inhalation of smoke from burning tobacco. Additionally, nonsmokers may also be exposed to these aldehydes indirectly through sidestream emissions, which are byproducts of smoking [20, 21].

These diverse sources necessitate an intricate understanding of their impact on public health. Aldehydes are significantly pervasive in our environment and show a close relationship with human health. In a recent study, Silva *et al*. [21] analyzed the 12 serum aldehydes in sera collected from 1843 participants in the 2013–2014 NHANES survey. Their data showed the widespread exposure of multiple types of aldehydes in the U.S. population, including isopentanaldehyde, propanaldehyde, butyraldehyde, heptanaldehyde, benzaldehyde, and hexanaldehyde. Different types of aldehydes can have distinct impacts on human health. Certain aldehydes, like formaldehyde, crotonaldehyde or hexanal, exhibit carcinogenic properties [22] or increase the risk of metabolic diseases [23]. In contrast, other aldehydes, such as cinnamaldehyde, have been demonstrated as protective factors against obesity, hyperglycemia, and non-alcoholic fatty liver disease [24, 25].

In our study, the weighted logistic regression analysis revealed a significant positive association between benzaldehyde concentration and CVD (OR = 1.17, 95% CI = 1.04–1.32) after adjusting for age, sex, race, education, hypertension, diabetes, smoking, and drinking. Benzaldehyde, a simple aromatic aldehyde, is prevalent both naturally and artificially [26]. It is found in many plant species, notably in bitter almond oil, and contributes to the characteristic almond scent. Benzaldehyde also results from environmental degradation processes, such as the breakdown of lignin, a key structural component in plant cell walls [27]. Besides its natural genesis, benzaldehyde is frequently synthesized for industrial use due to its appealing sweet aroma. Its applications are diverse, ranging from a flavor enhancer in food items and fragrance in personal care products to a precursor in synthesizing various organic compounds in chemical industries. Significantly, benzaldehyde is also present in vehicular exhaust and cigarette smoke, thus presenting a widespread exposure risk. Considering the pervasiveness of benzaldehyde, our results underscore the importance of managing benzaldehyde to mitigate its contribution to CVD development.

In a previous study by DeJarnett and colleagues, the epidemiological relationship between acrolein exposure and Framingham Risk Scores was investigated in 211 participants [28]. The findings indicated that acrolein exposure was linked to platelet activation and reduced levels of circulating angiogenic cells, thereby increasing the risk of CVD. Similarly, Liao *et al. * [13] investigated the association of benzaldehyde with CVD, discovering an increased risk (OR = 1.58, 95% CI = 1.15–2.17) at benzaldehyde concentrations >0.95 ng/mL. Additionally, compared with the lowest quartile (Q1), the Q2–Q4 isopentanaldehyde groups demonstrated increased ORs (95% CIs) of 1.48 (0.87, 2.52), 1.70 (1.01, 2.92), and 2.13 (1.19, 3.86), highlighting a graded relationship with CVD risk. However, their study did not account for the complex sampling design of NHANES survey, rendering their findings less robust. In contrast, our study incorporated survey weights according to the NHANES analytic guidance. We revealed a significant association between benzaldehyde and CVD but not in other aldehydes. The distinctive results are probably attributed to the difference in statistical analysis.

Our large-scale, nationally representative study demonstrated a positive relationship between benzaldehyde exposure and CVD. Nevertheless, several limitations should be mentioned. First, this study could only explore the association but not causality due to the cross-sectional continuous study design of NHANES survey. The following prospective study should be conducted to explore the longitudinal causal relationship. Second, there are various sources of aldehyde to explore. This study analyzed the serum aldehyde concentrations which suggested the overall exposure of aldehyde. However, the exact exposure sources of aldehyde were uncertain, thus limiting the clinical implication of this study. Third, the biological processes underlying the association between benzaldehyde exposure and CVD remains unclear. Forth, there exists a large difference in the number of patients between the two groups. Although we have applied the weighted statistics, the unbalanced distribution might result in potential bias. Last but not least, the outcome of this study is based on self-reported questionnaires. Although the interviewers have provided explanations for cardiovascular outcomes, the self-reported diseases might lead to inaccuracies in disease reporting. Therefore, our conclusion should be approached with caution. Further experiment analyses are warranted to elucidate the potential mechanisms underlying these associations.

## 5. Conclusions

In conclusion, this study highlights the potential role of benzaldehyde as a contributing factor to CVD. The observed association between benzaldehyde and CVD remained consistent in both unweighted and weighted logistic regression analyses, even after accounting for potential confounding factors such as age, sex, race, education, hypertension, diabetes, smoking and drinking. However, the study did not find a significant relationship between CVD risk and the concentrations of other examined aldehydes, including butyraldehyde, crotonaldehyde, decanaldehyde, heptanaldehyde, hexanaldehyde, isopentanaldehyde, nonanaldehyde, octanaldehyde, o-tolualdehyde, pentanaldehyde, and propanaldehyde. This indicates that the pathogenic effects of aldehydes on cardiovascular health may vary based on their distinct molecular structure and physicochemical properties. Future research should aim to validate these findings in diverse populations and delve deeper into understanding the biological mechanisms through which benzaldehyde may influence the development of CVD. Such studies will be crucial for developing targeted interventions and preventative strategies against CVD, particularly in the context of environmental and dietary exposure to specific aldehydes like benzaldehyde.

## Data Availability

The data for this study can be found on the NHANES website (https://www.cdc.gov/nchs/nhanes/index.htm).
